# Outdoor nitrogen dioxide exposure and longitudinal health status trajectory in the Canadian National Population Health Survey

**DOI:** 10.1038/s41598-024-79288-0

**Published:** 2024-12-28

**Authors:** David M. Stieb, Li Chen, Perry Hystad, Robyn Rittmaster, Eric Lavigne

**Affiliations:** 1 Environmental Health Science and Research Bureau, Health Canada, Vancouver, BC, Canada, Ottawa, ON Canada; 2 School of Epidemiology and Public Health, University of Ottawa, Ottawa, ON Canada; 3 College of Public Health and Human Sciences, Oregon State University, Corvallis, OR USA; 4 Risk Management Bureau, Health Canada, Ottawa, ON Canada

**Keywords:** Environmental impact, Diseases

## Abstract

Few studies have examined the association between air pollution and the trajectory of global health status measures related to the functional impacts of chronic disease. To address this gap, we examined the trajectory of the Health Utilities Index (HUI) over 17 years of follow-up among Canadian National Population Health Survey (NPHS) participants. Annual average nitrogen dioxide (NO_2_) exposures from a national land use regression surface were mapped to 15,631 NPHS participants at their place of residence provided at each follow-up. We modelled HUI trajectory as a cubic polynomial function of age in relation to air pollution and selected covariates using random growth curve models to account for longitudinal repeated measures. Adjusting for covariates selected based on a directed acyclic graph, we found that NO_2_ exposure exhibited a significant negative association with HUI in females. It also exhibited a significant positive interaction with the linear age term, and a significant negative interaction with the quadratic age term, resulting in a small non-significant decrease in quality adjusted life years lived after age 20 among females. Our analysis provides a proof of concept for examining the influence of built environment variables on the trajectory of health related quality of life in Canada.

## Introduction

The diverse adverse health effects of short- and long-term exposure to air pollution are well established^1^, including impacts on the incidence and severity of chronic disease^2–11^. However, few studies have examined the association between air pollution and the trajectory of physical functioning or other global health status measures related to the functional impacts of chronic disease, and results have been mixed^12–17^. Global health status measures are important because they provide a richer measure of disease impact than simply the presence or absence of disease. In Canada, previous studies have examined the trajectory of the Health Utilities Index (HUI), a multi-attribute measure of health-related quality of life (HRQOL) within the National Population Health Survey (NPHS)^18,19^. Ross et al. also examined neighbourhood determinants (socio-demographic characteristics and behavioural risk factors) of the HUI in Montreal, cross-sectionally using the Canadian Community Health Survey^20^. However, to our knowledge, no studies have evaluated associations of the trajectory of HRQOL with air pollution or other built environment features in Canada.

To address this gap, in the present study, we examined the trajectory of the HUI over 17 years of follow-up among NPHS participants. We hypothesized that HUI trajectory by age would diverge in relation to exposure to air pollution, reflecting an effective acceleration of the decline in health status by age. In contrast to measures examined in earlier studies, one highly desirable feature of the HUI is that it can be readily applied to quantify Quality Adjusted Life Years (QALYs)^21^. We provide an illustration of such an application based on our findings. More generally, our analysis provides a proof of concept for examining the influence of built environment variables on the trajectory of HRQOL in Canada.

## Results

Of 17,276 initial participants, 15,631 (90.8%) agreed to share their data with Health Canada. Of these, 6,863 (43.9%) participated in all nine cycles, 2,434 (15.6%) died, and 6,344 (40.5%) others did not complete all cycles. Sample characteristics are shown in Table 1. Study participants had an average age of 46.5 at cycle 1, with a slight preponderance of females over males, and incomes averaging approximately twice the low income cut-off. They were most commonly post-secondary graduates, mostly white by a large margin, non-smokers, physically inactive in their leisure time and not working in jobs requiring considerable physical effort. Place of residence was relatively stable, with a majority of participants reporting one or two postal codes over the duration of the study. Average nitrogen dioxide (NO_2_) concentrations over the duration of the study were highly correlated with estimated exposures at cycle 1 (Spearman ρ = 0.92).

**Table 1:** Sample characteristics.

Variable	Value	Frequency^a^ or mean	Percent or standard deviation
Age^b^	Years	46.5	17.7
Sex^b^	Male	6740	46.0
	Female	7925	54.0
Income ratio^c^		2.2	1.3
Highest Level of Education^d^	Less than high school	3570	24.3
	High school graduation	1865	12.7
	Some post-secondary	3175	21.7
	Post-secondary graduation	6060	41.3
	Missing	5	0.0
Racialized group membership^b^	White	13,785	94.0
	Chinese	205	1.4
	South Asian	170	1.2
	Indigenous^e^	160	1.1
	Black	165	1.1
	Other	185	1.3
Smoking^b^	Daily	3525	24.0
	Occasionally	620	4.2
	Not at all	9245	63.0
	Missing	1275	8.7
Physical Activity Index^b,f^	Active	2210	16.5
	Moderate	2600	19.4
	Inactive	6905	51.4
	Missing	830	6.2
Work requires a lot of physical effort^b^	Strongly agree	1140	14.1
	Agree	1805	22.3
	Neither agree nor disagree	560	6.9
	Disagree	2220	27.4
	Strongly disagree	580	7.2
	Missing	910	11.2
Number of postal codes	1	6020	41.1
	2	3965	27.0
	3	2275	15.5
	4 or more	2385	16.3
	Missing	20	0.1
Nitrogen dioxide^c^	Parts per billion	12.7	7.6
Health utilities index^b^		0.9	0.2

^a^In keeping with Statistics Canada data disclosure policy, counts are randomly rounded to base 5. ^b^Cycle 1, ^c^Mean – all cycles, ^d^Maximum – all cycles, ^e^Self-identifying as First Nations, Inuit or Métis, ^f^Derived from self-reported leisure time physical activity; active, moderate and inactive correspond to total daily energy expenditure ≥ 3, 1.5 to < 3, and < 1.5 kcal/kg/day respectively.

Regression model findings are summarized in Table 2. Although smoking was not included in the minimum adjustment set based on our DAG, its inclusion reduced the model AIC, thus it was retained. Conversely, physical activity was included in the minimum adjustment set, but its inclusion (leisure time physical activity alone or together with degree of physical exertion at work) did not reduce the model AIC, thus it was not retained. All coefficients were significantly different from 0 (p-value < 0.00001 to 0.012). Coefficients for age, age^2^ and age^3^ were consistent in magnitude modelled alone, together with NO_2_ and with NO_2_ and covariates. Coefficients for linear and cubic terms for age were negative, while the coefficient for the quadratic term was positive. Coefficients for NO_2_ were not sensitive to the addition of covariates, including physical activity (Supplementary Table S2 online). The coefficients for the main effect of NO_2_ and the interaction of NO_2_ with the quadratic age term were negative, while that for the interaction of NO_2_ with the linear term was positive. The coefficient for the NO_2_ main effect indicated that a decrease in NO_2_ concentration from the sample mean (12.7 ppb) to estimated natural background (0.15 ppb) would be associated with a 0.035 increase in HUI (95% CI 0.008, 0.063).

**Table 2:** Regression model coefficients by model specification. All models accounted for complex sampling design using bootstrap weights.

Variable	Base model		Base + NO_2_		Base + NO_2_ + covariates		
	Coefficient	p-value	Coefficient	p-value	Coefficient		p-value
Age	−2.08E−02	< 0.00001	−2.26E−02	< 0.00001	−2.49E−02		< 0.00001
Age^2^	5.28E−04	< 0.00001	5.48E−04	< 0.00001	6.03E−04		< 0.00001
Age^3^	−4.42E−06	< 0.00001	−4.41E−06	< 0.00001	−4.77E−06		< 0.00001
NO_2_			−3.44E−03	0.0027	−2.85E−03		0.012
NO_2_*Age			1.70E−04	< 0.00001	1.52E−04		< 0.00001
NO_2_*Age^2^			−1.88E−06	< 0.00001	−1.76E−06		< 0.00001
Sex					4.74E−02		0.0051
Sex*Age					−1.83E−03		< 0.00001
Sex*Age^2^					2.43E−05		< 0.00001
Sex*Age^3^					−1.41E−07		< 0.00001
Income ratio					−4.59E−02		< 0.00001
Income ratio*Age					4.12E−03		< 0.00001
Income ratio*Age^2^					−9.08E−05		< 0.00001
Income ratio*Age^3^					6.71E−07		< 0.00001
Less than high school					3.95E−01		< 0.00001
Less than high school*Age					−2.78E−02		< 0.00001
Less than high school*Age^2^					5.65E−04		< 0.00001
Less than high school*Age^3^					−3.49E−06		< 0.00001
Indigenous^a^					−2.59E−01		0.0014
Indigenous*Age					1.01E−02		< 0.00001
Indigenous*Age^2^					−1.08E−04		< 0.00001
Smoker					−3.11E−01		< 0.00001
Smoker*Age					2.21E−02		< 0.00001
Smoker*Age^2^					−4.95E−04		< 0.00001
Smoker*Age^3^					3.19E−06		< 0.00001

^a^Self-identifying as First Nations, Inuit or Métis.

**Fig. 1:**
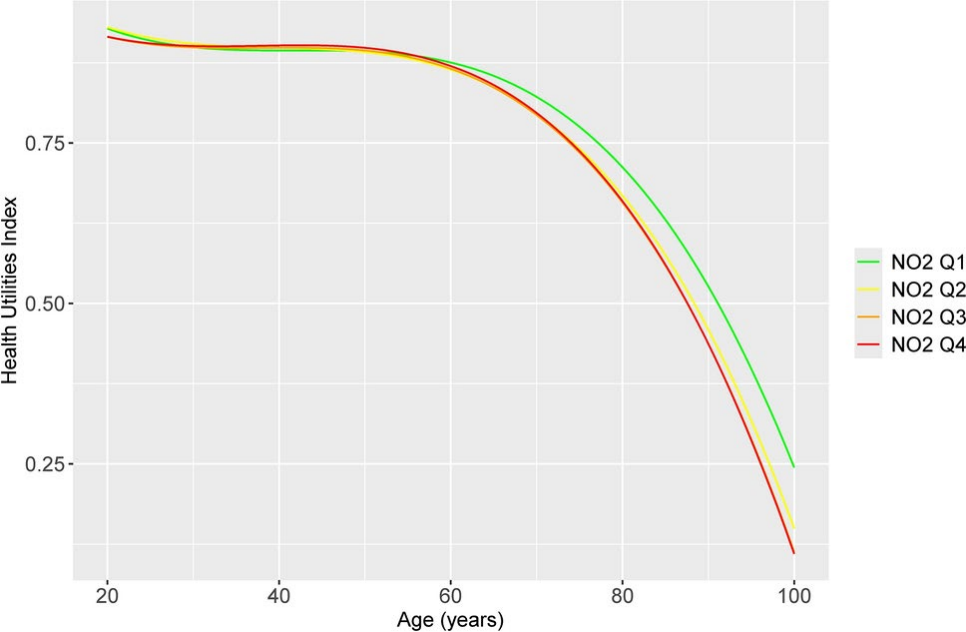
Predicted HUI trajectory by age and quartile of NO_2_ concentration, adjusting for sex, income, educational attainment, Indigenous identity and smoking and accounting for complex sampling design using bootstrap weights. The Figure shows the divergence of HUI trajectory with age comparing higher (red, orange and yellow lines) vs. lower (green line) NO_2_ exposure quartiles.

In sensitivity analyses (Supplementary Table S2 online), NO_2_ coefficients were smaller in magnitude compared to the base analysis and the NO_2_ main effect was no longer significant (p-value 0.44) when cycle 1 NO_2_ was substituted for average NO_2_ over the follow-up period and when analysis was restricted to participants with data from all nine cycles (p-value 0.40) and those reporting the same postal code over all nine cycles (p-value 0.92). Coefficients for NO_2_ and its interaction with age terms were similar to the base model in direction and relative magnitude in models employing transformed HUI values. Stratified analysis by urban vs. rural place of residence in cycle 1 revealed that coefficients for NO_2_, NO_2_*age and NO_2_* age^2^ were larger in magnitude for rural compared to urban residents, although the NO_2_ main effect coefficient was not statistically significant (p = 0.08). Models based on quartiles of NO_2_ exposure revealed that coefficients for the main effects of the third and fourth quartiles as well as their interactions with linear and quadratic age terms were larger in magnitude compared to those for the second quartile (Supplementary Fig. S3 online). The overall effect on the trajectory of HUI by age is shown in Fig. 1. The trajectories for all three of the higher quartiles clearly diverge from that of the first quartile, and at least partially conform to a clear gradient in that the Q2 downward trajectory with age is accelerated relative to Q1, and those of Q3 and Q4 (which overlap each other) are further accelerated relative to Q2. In addition, the AIC of the model employing a continuous NO_2_ variable was smaller than the model employing quartiles, indicating a better fit for the former.

In logistic regression models of survival and completion of all nine NPHS cycles, NO_2_, age, smoking, and Indigenous identity were associated with a reduction in the probability of completion of all cycles and survival, while female sex, income and education less than high school were associated with an increase in the probability of completion of all cycles and survival (Supplementary Table S4 online). Weighting of analyses using the inverse probability of completion of nine cycles based on the logistic regression model results reduced the magnitude of all coefficients and the main effect of NO_2_ was no longer significant (p-value 0.057). In contrast, the NO_2_ main effect coefficient increased in magnitude and statistical significance (p-value 0.0093) with weighting of analyses using the inverse probability of survival (Supplementary Table S2 online).

**Fig. 2:**
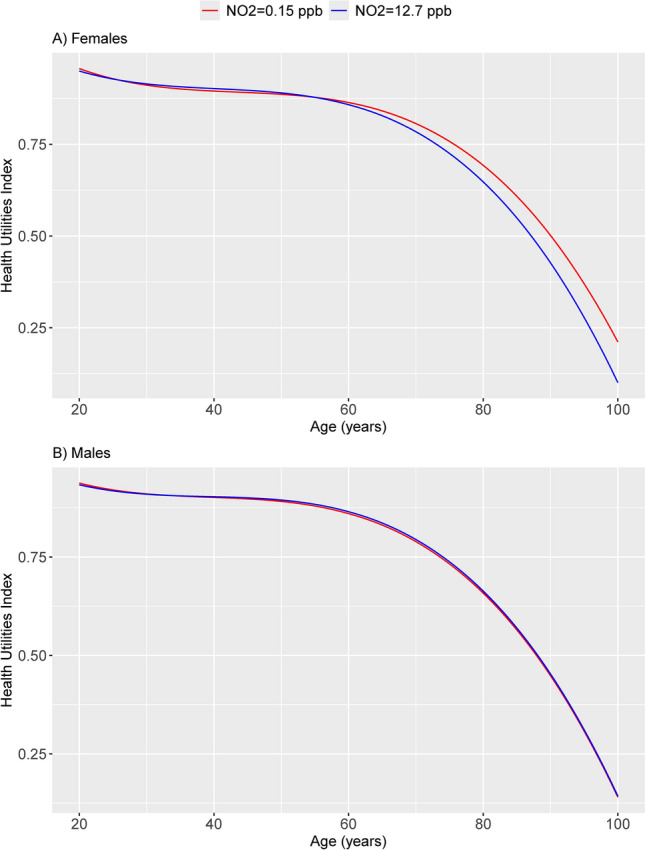
Predicted HUI trajectory by age and sample mean vs. estimated natural background NO_2_ concentration, adjusting for income, educational attainment, Indigenous identity and smoking and accounting for complex sampling design using bootstrap weights. The figure shows the divergence of HUI trajectory with age comparing higher (blue line) vs. lower (red line) NO_2_ exposure, in females (Panel **A**) but not males (Panel **B**).

In models stratified by sex, the NO_2_ main effect coefficient was significant for females (p-value 0.013) but not for males (p-value 0.54). Coefficients for interactions with linear and quadratic age terms were larger in magnitude for females than for males (Supplementary Fig. S5 online). Figure 2 presents the predicted HUI trajectory by age, sex and mean (12.7 ppb) vs. natural background NO_2_ concentration (0.15 ppb) based on the model adjusting for income, education, Indigenous identity and smoking. For females, based on the area under the curve from age 20 to 80, the model predicted 51.9 QALYs lived per person at background NO_2_ vs. 51.6 QALYs per person at the mean concentration (0.6% decrease, p-value 0.78). From age 20 to 100, the model predicted 61.6 QALYs lived at background NO_2_ vs. 59.8 QALYs at the mean concentration (2.9% decrease, p-value 0.75). QALYs were slightly larger based on the step method of quantifying the area under the HUI curve (e.g. 62.0 vs. 61.6 QALYs for both the spline and trapezoid methods, for females age 20 to 100 at natural background concentration and 60.2 vs. 59.8, for females age 20 to 100 at the mean concentration; the absolute differences being the same: 62.0–60.2 vs. 61.6–59.8 = 1.8 QALYs). Based on these findings, we estimated a gain of 4.7 million QALYs from age 20 to 80 in Canadian females from 2019 to 2079 comparing HUI trajectory by age based on NO_2_ exposure at the estimated background concentration vs. the observed mean concentration. For males, the HUI trajectories at higher vs. lower NO_2_ concentrations were virtually the same.

## Discussion

Adjusting for sex, income, educational attainment, Indigenous identity and smoking, we found that NO_2_ exposure exhibited a significant negative association with HUI. It also exhibited a significant positive interaction with the linear age term, and a significant negative interaction with the quadratic age term. The net effect was a modest acceleration in the decline of the HUI with age in females but not males, resulting in a small decrease in QALYs lived after age 20 among females, comparing average NO_2_ exposure to estimated natural background concentration.

The associations of air pollution with reduced HRQOL and its accelerated downward trajectory with age are biologically plausible. There is growing evidence, including from studies conducted in Canada, that air pollution exposure is associated with the incidence of several chronic conditions, including chronic obstructive pulmonary disease, ischemic heart disease, heart failure, diabetes, stroke, and dementia^2–4,6,9,22^. These conditions plausibly impact attribute domains of the HUI, particularly vision, speech, ambulation, dexterity, emotion, cognition, and pain. However, the specific etiological role of NO_2_ vs. other pollutants in the ambient air pollution mixture, remains to be determined^23^.

Few studies have examined the association between air pollution and trajectory of physical functioning or other global health status measures^13^. In their analysis of 5,708 participants in the Chicago Health and Aging Project (CHAP) study followed from 1993–2012, Weuve et al. found that NO_x_ was associated with more rapid progression of disability^17^. In contrast, de Zwart et al. found no association of air pollution (oxides of nitrogen and particulate matter) with rate of decline in physical functioning in 1,762 participants in the Longitudinal Aging Study Amsterdam (LASA) followed from 2005/6 to 2011/12^12^. They did however find a cross sectional association between air pollution and physical functioning^12^, as did Lin et al. in their analysis of impacts of PM_2.5_ in the Study on Global Ageing and Adult Health (SAGE) conducted among adults from China, Ghana, India, Mexico, Russia, and South Africa^15^. Myers et al. reported that long term PM_2.5_ exposure was associated with incident frailty in a study of 848 Israeli adults followed for 10–13 years after myocardial infarction^16^. Finally, Hu et al. found that proximity to major roadways was associated with physical disability in China^14^.

Interestingly, the NO_2_ main effect coefficient in our analysis was significant for females but not males. Lin et al. found greater cross sectional differences in physical functioning in association with air pollution in females^15^, while de Zwart found no sex or gender based differences^12^ and Weuve et al. did not examine differences in response by sex or gender^17^. In contrast, Hu et al. reported larger magnitude impacts in males^14^. More generally, findings regarding sex and gender as modifiers of the effects of air pollution are mixed^24^, thus this finding requires further evaluation.

Surprisingly, stratified analysis by urban vs. rural place of residence in cycle 1 revealed that coefficients for NO_2_, NO_2_*age and NO_2_* age^2^ were larger in magnitude for rural compared to urban residents, although the NO_2_ main effect coefficient was not statistically significant. While these findings should be interpreted with caution, as the sample size of rural residents was small, we hypothesize that this finding could be attributable to a combination of factors that would tend to increase exposure or susceptibility to health effects of air pollution, including greater average time spent outdoors, lower prevalence of air conditioning and a higher prevalence of smoking and chronic disease among rural vs. urban residents^25,26^.

Strengths of our study include the nationally representative sample, large sample size relative to earlier studies and long (17 year) follow-up. Unlike the CHAP and LASA studies which were restricted to single urban centres, the NPHS included urban and rural areas, with a sample size in 2010/11 twice that of CHAP with similar duration of follow-up, nearly seven times that of LASA with nearly three times the duration of follow-up, and 14 times that of the Myers et al. study. The NPHS included rich covariate data, and the HUI as a utility-theoretic HRQOL measure, which unlike earlier studies, can be directly applied to the estimation of QALYs lost or gained in the population. Quantification of changes in QALYs may also potentially be applied directly to economic evaluation of regulatory or other interventions to improve air quality^27^. Cost-effectiveness of a regulation is expressed as incremental cost per QALY gained (estimated incremental compliance costs relative to the status quo divided by QALYs gained e.g. $50 million additional compliance costs for a new program/1000 estimated QALYs gained as a result of the program = $50,000/QALY), also known as the incremental cost effectiveness ratio or ICER^28^. However, there is considerable debate on ICER thresholds for determining whether gains in QALYs are cost effective^29^. Moreover, while QALYs can capture impacts on both mortality and morbidity, estimates of willingness to pay for QALYs gained appear to differ depending on whether gains stem from changes in mortality or morbidity^30^. We also accounted for differential survival and attrition by NO_2_ exposure and participant characteristics. While the HUI is based on self-reported health status, it has been extensively validated in multiple settings, clinical contexts, countries and languages^31,32^. More generally, indices of health status/physical functioning based on self-report have been shown to predict future requirements for hospitalization and institutionalization^33^. Although covariate data (age, sex, income, educational attainment, racialized group membership, smoking and physical activity) were collected following best practices^34^, they were also based on self-report, which could introduce bias, the direction of which is difficult to predict. NO_2_ data were highly spatially resolved (30 m × 30 m), but estimated NO_2_ concentrations may not reflect changes in the spatial distribution of sources of NO_2_ over time. However, there is evidence that spatial gradients in traffic-related air pollutants such as NO_2_ tend to be stable over time^35–38^. While the sample was representative of the ethnic diversity of Canada’s population at the time of the cohort’s inception, it is no longer representative (9.4 percent of Canada’s population was characterized as “visible minority” at the time of the 1991 census^39^ in contrast to 25.6 percent based on the 2021 census^40^). Thus, findings may not be applicable to the current ethnic composition of the Canadian population. While we accounted for leisure time physical activity (based on estimated daily energy expenditure related to self-reported activities) and the degree of physical exertion at work, the latter was only crudely measured, and neither differentiated indoor vs. outdoor activity.

## Conclusions

In this analysis of the trajectory of HRQOL in a large, nationally representative Canadian cohort, we found that NO_2_ exposure was significantly associated with HRQOL, and exhibited significant interactions with age. The net effect was a non-significant decrease in QALYs lived after age 20 among females. Our analysis provides a proof of concept for examining the influence of built environment variables on the trajectory of HRQOL.

## Methods

### The National Population Health Survey

The National Population Health Survey (NPHS) is a nationally representative longitudinal survey of Canadians that was initiated in 1994 with a sample of 17,276 participants^41^. Follow-up with computer assisted interviews was conducted every two years, for a total of nine data collection cycles, completed in 2011. The initial response rate was 84.9% while the response rate for cycle 9 was 69.7% (cycle 9 respondents plus those who died or were institutionalized divided by 17,276)^41^. The NPHS includes rich data covering disability, diseases and health conditions, health status, use of health care services, health behaviours, social conditions, mental health and well-being, and prevention and detection of disease^41^.

### The health utilities index

Of particular interest in examining health trajectory longitudinally, each cycle of the NPHS collected data on the HUI, a validated general (as opposed to disease-specific) measure of HRQOL calibrated to a 0 to 1 scale, where 0 equals death and 1 equals perfect health (defined as the absence of limitations on all attribute domains)^21^. The HUI is a multi-attribute, utility-theoretic HRQOL measure that has been applied extensively in cost-utility analyses, particularly in health economic analysis of clinical care. It consists of two components: health state classification and rating of HRQOL. Health state classification is conducted across eight attribute domains, including vision, hearing, speech, ambulation, dexterity, emotion, cognition, and pain, each of which is scored according to five to six levels (e.g. for ambulation, six levels from “Able to walk around the neighbourhood without difficulty, and without walking equipment” to “Cannot walk at all”.)^21^. Domains are independent in that an individual with a given score on one domain can have any score on other domains^21^. Rating of HRQOL is accomplished by aggregating scores in each domain using a multiplicative multi-attribute utility function that accommodates interactions among domains^21^. Utility functions are models derived from empirical surveys in which respondents are asked to rate their preferences among health states described by diverse combinations of levels of the eight attribute domains. The NPHS collects data to classify each respondent’s health state at each survey cycle and then standard utility functions developed from these other surveys are used to calculate HUI values. QALYs are quantified as years of life multiplied by the HUI value (e.g. 1 year × 0.8 HUI = 0.8 QALYs). QALYs can therefore serve as an integrated measure of impacts on both mortality and morbidity.

### Nitrogen dioxide exposure

NO_2_ data were derived from a national land use regression (LUR) model incorporating ground monitoring data (from 134 government operated National Air Pollution Surveillance (NAPS) system fixed site monitors), remote sensing (using tropospheric NO_2_ columns from the Aura satellite together with the Goddard Earth Observing System—Chem chemical transport model to estimate ground-level concentrations), land use patterns (industrial land use within 2 km and length of road within 10 km), and weather (summer rainfall), with a resolution of 30 m × 30 m^42^. Deterministic gradients based on empirical studies were also added to account for local effects not captured by other variables. Specifically, NO_2_ concentrations were scaled up by factors of 1.65 and 1.2 fold adjacent to highways and major roads, decaying to 1 at distances of 300 m and 100 m respectively^42^. The NO_2_ model explained 73% of the variability in fixed-site monitor concentrations^42^. The LUR model was based on a single year (2006), then temporally scaled to other years based on ground monitoring data from NAPS monitoring stations for 24 Census Divisions^42^. These data have been employed extensively in large scale Canadian studies of a variety of health outcomes^2,4,6,43,44^. Annual average NO_2_ exposures from this surface were mapped to NPHS participants by six character postal code at their place of residence provided at each cycle. Alternative exposure windows (baseline i.e. NPHS cycle 1, period average i.e. averaged over all NPHS cycles) were considered.

### Statistical analysis

Statistical analysis of HUI trajectory by age in relation to air pollution and selected covariates employed random growth curve models to account for longitudinal repeated measures^18,19,45^. These models permit a variety of non-linear functional forms and treat NPHS participants as random effects, allowing intercepts and/or slopes to vary randomly among participants. Specifically, since there are multiple data points over time for each participant, different intercepts and/or slopes may be estimated for each participant, rather than assuming the same intercept and slope for all participants. In keeping with Ross et al., we modelled HUI as a cubic polynomial function of age as shown in Eq. 1

1 $${HUI}_{i}={\beta }_{0}+{\beta }_{1}\times {age}_{i}+{\beta }_{2}\times {{age}_{i}^{2}+\beta }_{3}\times {age}_{i}^{3}+\sum {\beta }_{j}\times {x}_{j}+\sum ({\beta }_{k}\times {age}_{i}\times {x}_{j}+{\beta }_{l}\times {age}_{i}^{2}\times {x}_{j}+{\beta }_{m}\times {age}_{i}^{3}\times {x}_{j})$$

where ${x}_{j}$ represents a vector of covariates, including NO_2_. Covariates were selected based on the directed acyclic graph^46^ shown in Supplementary Fig. S1 online. HUI measures HRQOL across multiple functional domains; therefore we identified the primary determinants of HUI as chronic disease and age, independent of chronic disease, since functional limitations are expected to arise with age even in the absence of chronic disease. We conceptualized air pollution/ built environment exposure, physical activity and smoking as the three other primary determinants of chronic disease. We specified a bi-directional relationship between air pollution/built environment and physical activity, in that the quality of the built environment (including air pollution exposure) influences the likelihood of physical activity, while (outdoor) physical activity increases exposure to air pollution and the built environment. Finally, personal characteristics (age, sex, socioeconomic status and racialized group membership) were conceptualized as influencing air pollution/built environment exposure, physical activity, incidence of chronic disease and smoking, while age, sex and racialized group membership are determinants of socioeconomic status. The DAG indicates that the minimum covariate adjustment set includes age, sex, socioeconomic status, physical activity and racialized group membership. Two options were considered to represent physical activity: leisure time physical activity alone and combined with degree of physical exertion at work. The leisure time physical activity index classified individuals as active, moderate and inactive based on total daily energy expenditure ≥ 3, 1.5 to < 3, and < 1.5 kcal/kg/day respectively based on self-reported activities. Work-related physical exertion was based on response to the statement, “Work requires a lot of physical effort” on a five point scale from “strongly agree” to “strongly disagree”. Whole number scores representing leisure time and work physical activity were first averaged separately over all NPHS cycles, then added together for a combined average score. Those NPHS participants who did not work were assigned a score of zero for physical exertion at work. Models were built using the R package, lme4^47^ and the SAS Mixed procedure, retaining covariates and their interactions with age terms if their addition reduced the model Akaike Information Criterion (AIC)^48^. Once the best-fitting model was established, the complex sampling design was accounted for employing Bootstrap weights^41^. We conducted a sensitivity analysis employing a transformed HUI (arcsine [{2 × {(HUI + 0.36)/(1 + 0.36)}} − 1]), which Ross et al. found improved the distribution of model residuals^19–21^, though we retained the untransformed HUI values as our base model for ease of interpretation. As an additional sensitivity analysis, we accounted for differential attrition by NO_2_ exposure and participant characteristics following Weuve et al. and de Zwart et al.^12,17^. We constructed logistic regression models of survival and completion of all nine NPHS cycles with predictors comprising the same variables as our models of HUI. Inverse probabilities of survival and completion of all cycles were then employed as weights in models of HUI by age, effectively giving greater weight to participants with a lower probability of survival or completion of all cycles. The shape of the NO_2_-HUI concentration–response relationship was examined by specifying exposure by quartile, similar to previous studies^12,17^.

The impact of air pollution on QALYs lived per person was quantified as the difference in the area under the curve of HUI by age from age 20 (the minimum age of participants included in our analysis) to ages 80 and 100 (the approximate maximum age in all NPHS cycles) for an increment in NO_2_ from the estimated natural background concentration (0.15 ppb)^49^ to the sample mean from our analysis. Area under the curve was estimated using the AUC function in the R DescTools package^50^, with the method specified as spline. The AUC function specifies a natural cubic spline fit through three times the number of data points provided by the user (in this case, 3*61 and 3*81 points for the HUI curves by age from 20 to 80 and 100 respectively). We also conducted sensitivity analyses employing the “trapezoid” and “step” methods of determining area under the curve. As the names suggest, these methods approximate the total AUC as the sum of the area of a series of trapezoids and rectangles, respectively. To quantify QALY impacts at the population level, estimated population counts by single year of age were obtained for 2019. Probabilities of survival by single year of age were obtained from the 2019 Canadian life table^51^ in order to estimate the surviving population in each age group to age 80 and 100. These estimates of QALYs gained are conservative in that they are derived solely from the 2019 population as it ages, without accounting for net population growth through birth and immigration over the 60–80 year time span of the analysis. Although population and survival probability estimates were available for more recent years, we selected 2019 in order to avoid impacts of the COVID-19 pandemic on these parameters. Uncertainty was captured using a Monte Carlo simulation sampling from the distribution of NO_2_ regression coefficients using 1,000 iterations.

Statistical analyses were conducted in R version 4.1.1 and SAS Enterprise Guide 7.1. All tests of statistical significance were two-tailed.

This study was approved by the Health Canada Research Ethics Board (#2022-020H). NPHS data were collected in accordance with the Tri-Council Policy on Ethical Conduct for Research Involving Humans^52^ as well as the Statistics Act^53^, and informed consent was obtained from all participants and/or their legal guardians.

## Supplementary Information

Supplementary Information.

## Data Availability

The NPHS data that support the findings of this study are available from Statistics Canada (https://www.statcan.gc.ca/en/start), but restrictions apply to the availability of these data, which were used under license for the current study. The NO2 data employed in this study are publicly available from the Canadian Urban Environmental Health Research Consortium (https://canue.ca/).

## References

[1] Thurston, G. D. et al. A joint ERS/ATS policy statement: What constitutes an adverse health effect of air pollution? An analytical framework. *Eur. Respir. J.* **49**, 1600419 (2017). 28077473 10.1183/13993003.00419-2016 PMC5751718

[2] Bai, L. et al. Exposure to ambient air pollution and the incidence of congestive heart failure and acute myocardial infarction: A population-based study of 5.1 million Canadian adults living in Ontario. *Environ. Int.* **132**, 105004 (2019). 31387019 10.1016/j.envint.2019.105004

[3] Chen, H. et al. Living near major roads and the incidence of dementia, Parkinson’s disease, and multiple sclerosis: A population-based cohort study. *Lancet* **389**, 718–726 (2017). 28063597 10.1016/S0140-6736(16)32399-6

[4] Olaniyan, T. et al. Ambient air pollution and the risk of acute myocardial infarction and stroke: A national cohort study. *Environ. Res.* **204**, 111975 (2022). 34478722 10.1016/j.envres.2021.111975

[5] Rugel, E. J. & Brauer, M. Quiet, clean, green, and active: A Navigation Guide systematic review of the impacts of spatially correlated urban exposures on a range of physical health outcomes. *Environ. Res.* **185**, 109388 (2020). 32244108 10.1016/j.envres.2020.109388

[6] Shin, S. et al. Air pollution as a risk factor for incident chronic obstructive pulmonary disease and asthma. A 15-year population-based cohort study. *Am. J. Respir. Crit. Care Med.* **203**, 1138–1148 (2021). 33147059 10.1164/rccm.201909-1744OC

[7] To, T. et al. Chronic disease prevalence in women and air pollution—A 30-year longitudinal cohort study. *Environ. Int.* **80**, 26–32 (2015). 25863281 10.1016/j.envint.2015.03.017

[8] To, T. et al. Progression from asthma to chronic obstructive pulmonary disease. Is air pollution a risk factor?. *Am. J. Respir. Crit. Care Med.* **194**, 429–438 (2016). 26950751 10.1164/rccm.201510-1932OC

[9] Weichenthal, S. et al. Long-term exposure to ambient ultrafine particles and respiratory disease incidence in in Toronto, Canada: A cohort study. *Environ. Health Glob. Access Sci. Source* **16** (2017). 10.1186/s12940-017-0276-7 PMC5477122 28629362

[10] Yankoty, L. I. et al. Long-term residential exposure to environmental/transportation noise and the incidence of myocardial infarction. *Int. J. Hyg. Environ. Health* **232**, 113666 (2021). 33296779 10.1016/j.ijheh.2020.113666

[11] Zhang, Z. et al. A population-based cohort study of respiratory disease and long-term exposure to iron and copper in fine particulate air pollution and their combined impact on reactive oxygen species generation in human lungs. *Environ. Sci. Technol.* **55**, 3807–3818 (2021). 33666410 10.1021/acs.est.0c05931

[12] de Zwart, F., Brunekreef, B., Timmermans, E., Deeg, D. & Gehring, U. Air pollution and performance-based physical functioning in Dutch older adults. *Environ. Health Perspect.* **126**, 017009 (2018). 29364820 10.1289/EHP2239 PMC6014703

[13] Garcia-Esquinas, E. & Rodriguez-Artalejo, F. Environmental pollutants, limitations in physical functioning, and frailty in older adults. *Curr. Environ. Health Rep.* **4**, 12–20 (2017). 28091981 10.1007/s40572-017-0128-1

[14] Hu, J. et al. Residential proximity to major roadway and progression in physical disability in older adults in China. *Environ. Sci. Pollut. Res.* **29**, 36616–36625 (2022). 10.1007/s11356-021-18203-w 35064490

[15] Lin, H. et al. Exposure to ambient PM_2.5_ associated with overall and domain-specific disability among adults in six low- and middle-income countries. *Environ. Int.* **104**, 69–75 (2017). 28453972 10.1016/j.envint.2017.04.004

[16] Myers, V. et al. Exposure to particulate air pollution and long-term incidence of frailty after myocardial infarction. *Ann. Epidemiol.* **23**, 395–400 (2013). 23790344 10.1016/j.annepidem.2013.05.001

[17] Weuve, J. et al. Exposure to traffic-related air pollution in relation to progression in physical disability among older adults. *Environ. Health Perspect.* **124**, 1000–1008 (2016). 27022889 10.1289/ehp.1510089 PMC4937863

[18] Orpana, H. M. et al. The natural history of health-related quality of life: A 10-year cohort study. *Health Rep.* **20**, 29–35 (2009). 19388366

[19] Ross, N. A. et al. Trajectories of health-related quality of life by socio-economic status in a nationally representative Canadian cohort. *J. Epidemiol. Community Health* **66**, 593–598 (2012). 21441176 10.1136/jech.2010.115378 PMC3560850

[20] Ross, N. A., Tremblay, S. S. & Graham, K. Neighbourhood influences on health in Montreal, Canada. *Soc. Sci. Med.* **1982**(59), 1485–1494 (2004). 10.1016/j.socscimed.2004.01.016 15246176

[21] Feeny, D. et al. Multiattribute and single-attribute utility functions for the health utilities index mark 3 system. *Med. Care* **40**, 113–128 (2002). 11802084 10.1097/00005650-200202000-00006

[22] Chen, H. et al. Risk of incident diabetes in relation to long-term exposure to fine particulate matter in Ontario, Canada. *Environ. Health Perspect.* **121**, 804–810 (2013). 23632126 10.1289/ehp.1205958 PMC3701997

[23] Boogaard, H. et al. Long-term exposure to traffic-related air pollution and selected health outcomes: A systematic review and meta-analysis. *Environ. Int.* **164**, 107262 (2022). 35569389 10.1016/j.envint.2022.107262

[24] Zhang, J. et al. Sex differences in cardiovascular risk associated with long-term PM_2.5_ exposure: A systematic review and meta-analysis of cohort studies. *Front. Public Health* **10**, 802167 (2022). 35186842 10.3389/fpubh.2022.802167 PMC8847390

[25] Stieb, D. M. et al. Variability in ambient ozone and fine particle concentrations and population susceptibility among Canadian health regions. *Can. J. Public Health.* **110**, 149–158 (2019). 30617991 10.17269/s41997-018-0169-8 PMC6964403

[26] Matz, C. J., Stieb, D. M. & Brion, O. Urban-rural differences in daily time-activity patterns, occupational activity and housing characteristics. *Environ. Health Glob. Access Sci. Source* **14** (2015). 10.1186/s12940-015-0075-y PMC4644325 26566986

[27] Krutilla, K., Good, D. H. & Graham, J. D. Uncertainty in the cost-effectiveness of federal air quality regulations. *J. Benefit-Cost Anal.* **6**, 66–111 (2015).

[28] Canadian Agency for Drugs and Technologies in Health. *Guidelines for the Economic Evaluation of Health Technologies: Canada (4th Edition)* (Ottawa, Canada, 2017).

[29] Brouwer, W., van Baal, P., van Exel, J. & Versteegh, M. When is it too expensive? Cost-effectiveness thresholds and health care decision-making. *Eur. J. Health Econ.* **20**, 175–180 (2019). 30187251 10.1007/s10198-018-1000-4

[30] Ryen, L. & Svensson, M. The willingness to pay for a quality adjusted life year: A review of the empirical literature. *Health Econ.* **24**, 1289–1301 (2015). 25070495 10.1002/hec.3085

[31] Furlong, W. J., Feeny, D. H., Torrance, G. W. & Barr, R. D. The Health Utilities Index (HUI®) system for assessing health-related quality of life in clinical studies. *Ann. Med.* **33**, 375–384 (2001). 11491197 10.3109/07853890109002092

[32] Miller, W., Robinson, L. & Lawrence, R. *Valuing Health for Regulatory Cost-Effectiveness Analysis* (The National Academies Press, 2006).

[33] Guralnik, J. M. et al. A short physical performance battery assessing lower extremity function: Association with self-reported disability and prediction of mortality and nursing home admission. *J. Gerontol.* **49**, M85-94 (1994). 8126356 10.1093/geronj/49.2.m85

[34] Statistics Canada. *Survey Methods and Practices*. https://www150.statcan.gc.ca/n1/en/pub/12-587-x/12-587-x2003001-eng.pdf?st=tjeTGsWA (2010).

[35] Cesaroni, G. et al. Nitrogen dioxide levels estimated from land use regression models several years apart and association with mortality in a large cohort study. *Environ. Health* **11**, 48 (2012). 22808928 10.1186/1476-069X-11-48 PMC3407745

[36] de Hoogh, K. et al. Spatial PM_2.5_, NO_2_, O_3_ and BC models for Western Europe—Evaluation of spatiotemporal stability. *Environ. Int.* **120**, 81–92 (2018). 30075373 10.1016/j.envint.2018.07.036

[37] Eeftens, M. et al. Stability of measured and modelled spatial contrasts in NO_2_ over time. *Occup. Environ. Med.* **68**, 765–770 (2011). 21285243 10.1136/oem.2010.061135

[38] Wang, R., Henderson, S. B., Sbihi, H., Allen, R. W. & Brauer, M. Temporal stability of land use regression models for traffic-related air pollution. *Atmos. Environ.* **64**, 312–319 (2013).

[39] Statistics Canada. Census: Ethnocultural portrait: Sub-provincial. https://www12.statcan.gc.ca/English/census01/products/analytic/companion/etoimm/subprovs.cfm.

[40] Statistics Canada. Visible minority by gender and age: Canada, provinces and territories. https://www150.statcan.gc.ca/t1/tbl1/en/tv.action?pid=9810035101 (2023).

[41] Statistics Canada. *National Population Health Survey Household Component Cycles 1 to 9 (1994/1995 To 2010/2011) Longitudinal Documentation* (2012).

[42] Hystad, P. et al. Creating national air pollution models for population exposure assessment in Canada. *Environ. Health Perspect.* **119**, 1123–1129 (2011). 21454147 10.1289/ehp.1002976 PMC3237350

[43] Crouse, D. L. et al. Ambient PM_2.5_, O_3_, and NO_2_ exposures and associations with mortality over 16 years of follow-up in the Canadian census health and environment cohort (CanCHEC). *Environ. Health Perspect.* **123**, 1180–1186 (2015). 26528712 10.1289/ehp.1409276 PMC4629747

[44] Stieb, D. M. et al. A national study of the association between traffic-related air pollution and adverse pregnancy outcomes in Canada, 1999–2008. *Environ. Res.* **148**, 513–526 (2016). 27155984 10.1016/j.envres.2016.04.025

[45] Mirman, D. *Growth Curve Analysis and Visualization Using R* (CRC Press, 2014).

[46] Textor, J., van der Zander, B., Gilthorpe, M. S., Liśkiewicz, M. & Ellison, G. T. H. Robust causal inference using directed acyclic graphs: The R package ‘dagitty’. *Int. J. Epidemiol.* **341**, 1887–1894. 10.1093/ije/dyw341 (2017). 10.1093/ije/dyw341 28089956

[47] Bates, D., Maechler, M., Bolker, B. & Walker, S. Fitting linear mixed-effects models using lme4. *J. Stat. Softw.* **67**, 1–48 (2015).

[48] Akaike, H. A new look at the statistical model identification. *IEEE Trans. Autom. Control* **19**, 716–723 (1974).

[49] Health Canada. *Health Impacts of Air Pollution in Canada: Estimates of Premature Deaths and Nonfatal Outcomes - 2021 Report.* (2021).

[50] Signorell, A. *R Package ‘DescTools’: Tools for Descriptive Statistics (Version 0.99.41)*. https://cran.r-project.org/package=DescTools (2021).

[51] Statistics Canada. Life expectancy and other elements of the complete life table, single-year estimates, Canada, all provinces except Prince Edward Island. https://www150.statcan.gc.ca/t1/tbl1/en/tv.action?pid=1310083701 (2022).

[52] Government of Canada, Interagency Advisory Panel on Research Ethics. Tri-Council Policy Statement: Ethical Conduct for Research Involving Humans – TCPS 2 (2022). https://ethics.gc.ca/eng/policy-politique_tcps2-eptc2_2022.html (2023).

[53] Legislative Services Branch. Consolidated federal laws of Canada, Statistics Act. https://laws-lois.justice.gc.ca/eng/acts/S-19/FullText.html (2017).

