# Supplementary material for: Outdoor nitrogen dioxide exposure and longitudinal health status trajectory in the Canadian National Population Health Survey
Source: Sci Rep. 2024 Dec 28;14:30746. doi: 10.1038/s41598-024-79288-0 (PMC11681082; doi:10.1038/s41598-024-79288-0)

Supplementary Information


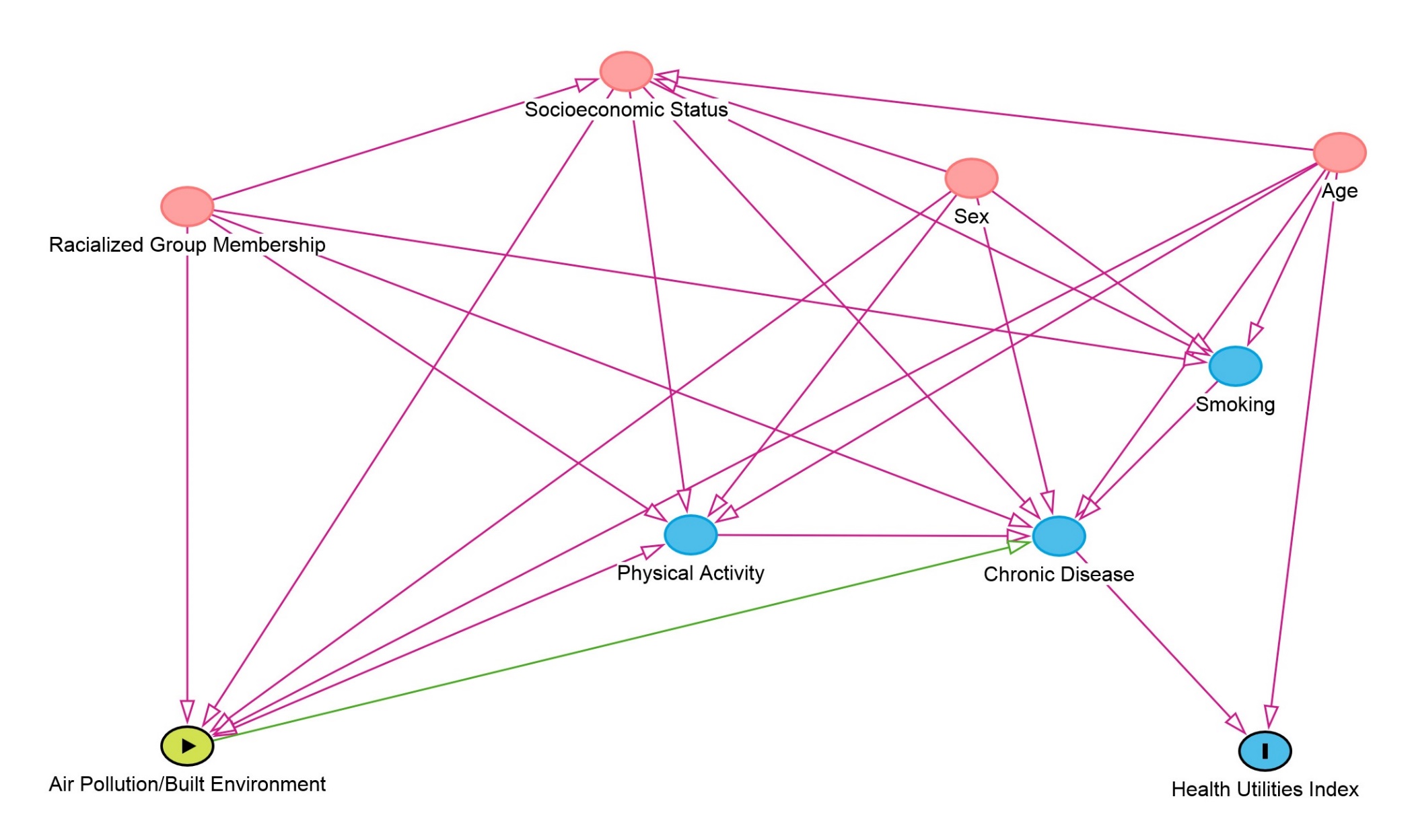


Supplementary Figure S1. Directed acyclic graph of putative causal (green) and biasing (purple) pathways between exposures of interest (air pollution, built environment) and outcome measure (health utilities index). Red ovals indicate ancestors of exposure and outcome; blue ovals indicate ancestor of outcome.

Supplementary Table S2. Regression model coefficients for NO_2_ main effects and interactions with age - base and sensitivity analyses. All models adjusted for sex, income, educational attainment, Indigenous identity and smoking and accounted for complex sampling design using bootstrap weights.

|  | NO_2_ | | NO_2_*Age | | NO_2_*Age^2^ | |
| --- | --- | --- | --- | --- | --- | --- |
|  | Coefficient | p-value | Coefficient | p-value | Coefficient | p-value |
| Base Analysis | -2.85E-03 | 0.012 | 1.52E-04 | <0.00001 | -1.76E-06 | <0.00001 |
| Covariate for leisure time physical activity | -2.86E-03 | 0.016 | 1.58E-04 | <0.00001 | -1.85E-06 | <0.00001 |
| Covariate for combined leisure time and work physical activity | -3.13E-03 | 0.0083 | 1.66E-04 | <0.00001 | -1.88E-06 | <0.00001 |
| Rural (n=3024) | -1.10E-02 | 0.080 | 6.79E-04 | <0.00001 | -9.19E-06 | <0.00001 |
| Urban (n=10073) | -6.86E-04 | 0.61 | 6.49E-05 | <0.00001 | -9.17E-07 | <0.00001 |
| Transformed HUI | -8.99E-03 | <0.00001 | 3.77E-04 | <0.00001 | -3.73E-06 | <0.00001 |
| Cycle 1 NO_2_ | -7.60E-04 | 0.44 | 4.50E-05 | <0.00001 | -5.54E-07 | <0.00001 |
| Participated All Cycles (n=6077) | -1.67E-03 | 0.40 | 8.97E-05 | <0.00001 | -1.14E-06 | <0.00001 |
| Same Postal Code All Cycles (n=2193) | -1.75E-04 | 0.92 | 2.19E-05 | 0.021 | -2.46E-07 | 0.0014 |
| Completion Inverse Probability Weighting | -2.17E-03 | 0.057 | 9.88E-05 | <0.00001 | -1.08E-06 | <0.00001 |
| Survival Inverse Probability Weighting | -3.19E-03 | 0.0093 | 1.59E-04 | <0.00001 | -1.73E-06 | <0.00001 |

Supplementary Figure S3. Regression coefficients and 95% confidence intervals for NO_2_ main effects and interactions with linear and quadratic age terms, from model employing NO_2_ quartiles


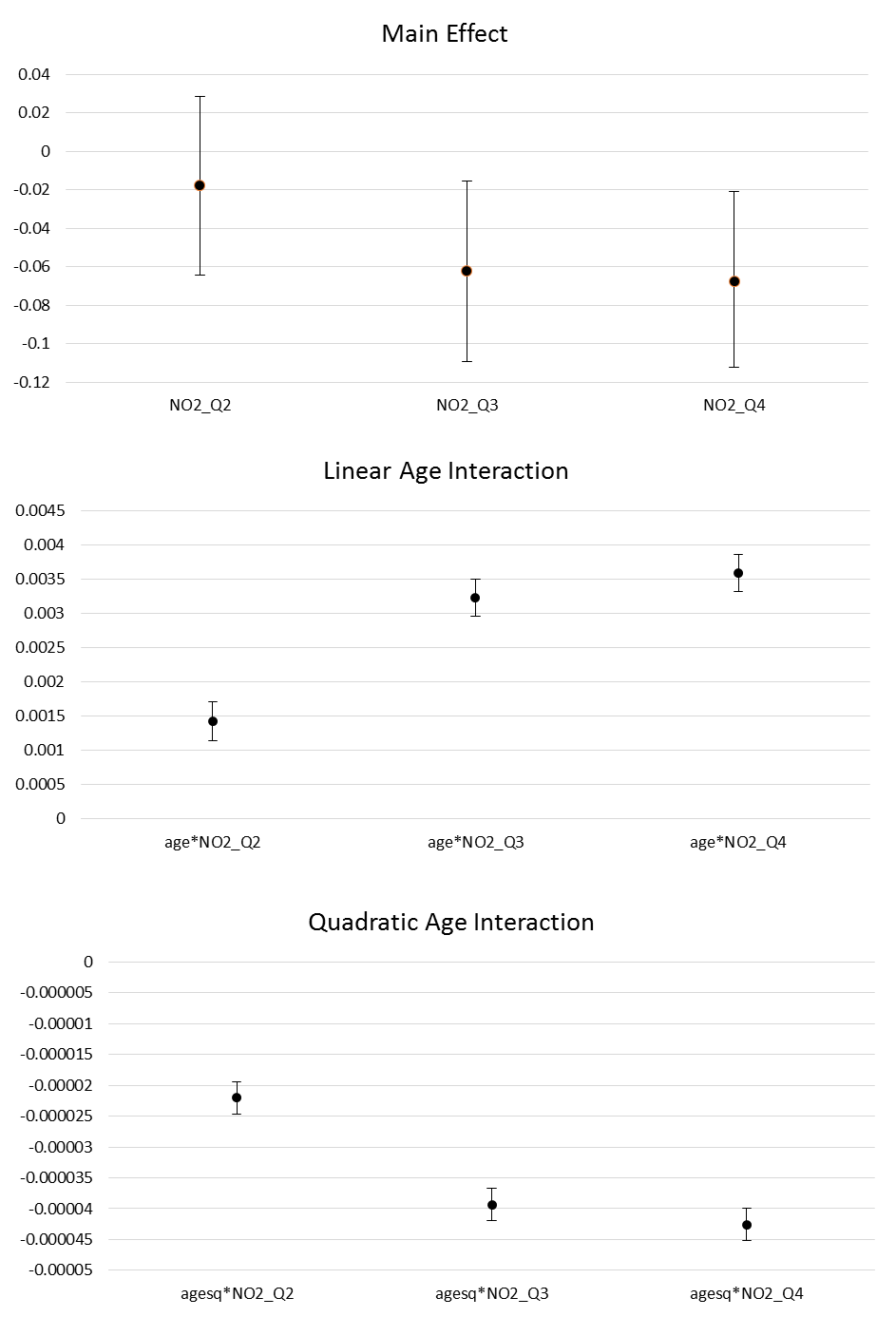


Supplementary Table S4. Odds ratios from logistic regression models of completion of all cycles and survival

| Variable | Odds Ratio  (95% confidence interval) | |
| --- | --- | --- |
|  | Completion of Nine NPHS Cycles | Survival |
| Age (10 year increment) | 0.86 (0.84-0.88) | 0.29 (0.28-0.31) |
| Sex (female vs. male) | 1.45 (1.34-1.56) | 1.94 (1.69-2.22) |
| Income Ratio (1 unit increment) | 1.36 (1.31-1.4) | 1.27 (1.19-1.36) |
| Less than high school vs. high school or greater | 1.68 (1.51-1.85) | 1.19 (1.03-1.36) |
| Indigenous vs. non-Indigenous | 0.33 (0.21-0.52) | 0.56 (0.27-1.14) |
| Daily or occasional smoker vs. non-smoker | 0.71 (0.66-0.77) | 0.38 (0.32-0.44) |
| Mean NO_2_ (0.15 to 12.7 ppb increment) | 0.56 (0.53-0.60) | 0.84 (0.75-0.93) |

Supplementary Figure S5. Regression coefficients and 95% confidence intervals for NO_2_ main effects and interactions with linear and quadratic age terms, for females and males


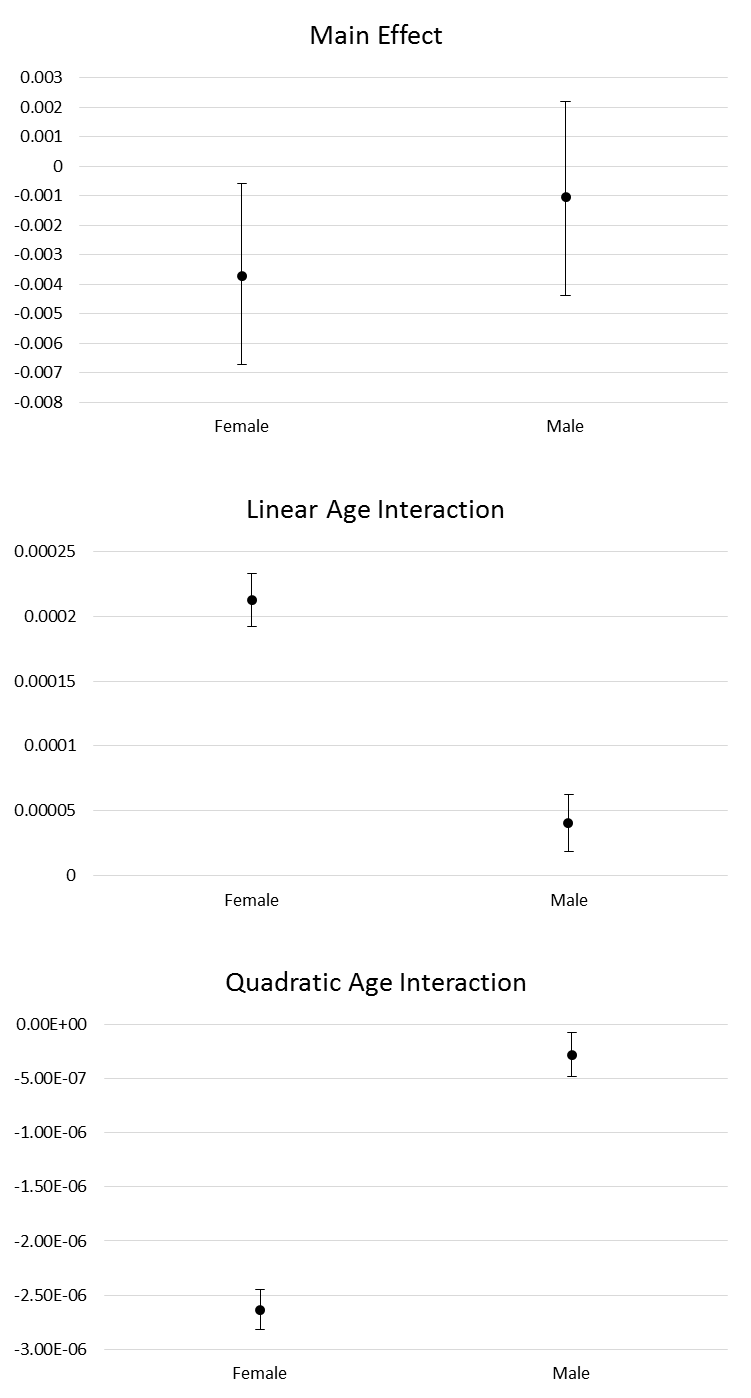

Supplement: Supplementary file 1 — Supplementary Information. [file 41598_2024_79288_MOESM1_ESM.docx]
